# Who thrives in Canada? An Examination of social factors, healthcare access, and immigration status

**DOI:** 10.1371/journal.pgph.0005257

**Published:** 2025-12-04

**Authors:** Sonia S. Anand, Shreni Patel, Scott A. Lear, Trevor J.B. Dummer, Vikki Ho, Jean-Claude Tardif, Jennifer E. Vena, Karleen Schulze, Paul Poirier, Dipika Desai, Matthias G. Friedrich, from the Canadian Alliance for Healthy Hearts and Minds Study

**Affiliations:** 1 Chanchlani Research Centre, Department of Medicine, McMaster University, Hamilton, Ontario, Canada; 2 Mary Heersink School of Global Health and Social Medicine, Faculty of Health Sciences, McMaster University, Hamilton, Ontario, Canada; 3 Population Health Research Institute, Hamilton Health Sciences, Hamilton, Ontario, Canada; 4 Faculty of Health Sciences, Simon Fraser University, Burnaby, British Columbia, Canada; 5 School of Population and Public Health, University of British Columbia, Vancouver, British Columbia, Canada; 6 Université de Montréal Hospital Research Centre (CRCHUM), Université de Montréal, Montreal, Quebec, Canada; 7 Department of Social and Preventive Medicine, Université de Montréal, Montreal, Quebec, Canada; 8 Department of Medicine, Montreal Heart Institute, Université de Montréal, Montreal, Quebec, Canada; 9 Alberta’s Tomorrow Project, Cancer Research & Analytics, Cancer Care Alberta, Alberta Health Services, Calgary, Alberta, Canada; 10 Department of Medicine, McMaster University, Hamilton, Ontario, Canada; 11 Faculté de pharmacie, Université Laval, Institut universitaire de cardiologie et de pneumologie de Québec, Québec City, Quebec, Canada; 12 Departments of Cardiology and Diagnostic Radiology, McGill University Health Center, Montréal, Quebec, Canada; University of New Brunswick, CANADA

## Abstract

High-income countries like Canada report some of the worlds’ highest life-satisfaction levels, yet less is known about how life satisfaction varies by race and immigration status. This study investigates the factors that influence subjective well-being among 8,063 adults from the Canadian Alliance of Healthy Hearts and Minds study recruited between 2014 and 2018, including a subset of 2,142 immigrants. Measures of demographic, socioeconomic, health, healthcare access, and self-reported ethnicity were investigated in relation to self-reported life satisfaction as measured by the validated Cantril ladder score in which people were classified as suffering [1–4], struggling [5–6], or thriving [7–10]. Among 8,063 adults, approximately half were women, 18.6% were racialized, and 26.6% were immigrants. The mean life satisfaction score was 7.2 (1.4), with 71% classified as thriving. However racialized immigrants reported significantly lower life satisfaction than Canadian born non-racialized participants [6.6 (1.6) vs 7.2 (1.4); P < 0.001, and a lower proportion were classified as thriving [57% vs 73%]. In the overall sample, multivariable linear regression showed higher life satisfaction was associated with older age, male sex, having trusted neighbours, and having a language-concordant family doctor. Lower life satisfaction was associated with social disadvantage, being female, having poorer cardiovascular health, being unable to afford prescription medications, seeking care in an emergency department, and being racialized. Amongst the subset of immigrants, the life satisfaction associated factors were directionally consistent and racialized immigrants reported lower life satisfaction due to discrimination based on skin colour. Although Canada has amongst the highest life-satisfaction scores globally, the average masks persistent inequities as racialized people (especially racialized immigrants) have lower life satisfaction than non-racialized people. The findings highlight actionable levers—language-concordant primary care attachment, affordable medications, neighbourhood trust, and improved cardiometabolic health—that can be targeted to close the observed well-being gap.

## Introduction

Life satisfaction is a global indicator of subjective well-being that has been evaluated globally. Canada ranks highly in comparison to the 156 countries included in the World Happiness Report [1]. The validated Cantril Ladder Score provides a simple, widely used measure of current life satisfaction and expectations about the future [2], and is strongly associated with health status, health behaviours [3–5], and chronic diseases, including cardiovascular disease [6–9]. Gross Domestic Product (GDP) per capita, social support, freedom to make choices, healthy life expectancy, generosity, and absence of corruption, explain three-quarters of the variation between countries [10].

Despite Canada’s comparatively high national average score and 9^th^ place rank globally in 2019, within Canada variability of life satisfaction scores could exist and understanding its determinants remains policy relevant [10]. Prior literature from Canada shows that social determinants such as employment, income, education, housing, and experiences of racism shape both health and subjective well-being [11]. Although Canada’s 1984 Canada Health Act strives for equal healthcare access, barriers including limited primary care attachment, language discordance, complex system navigation, and institutional racism persist [12,13], Furthermore, Canada welcomes a substantial proportion immigrants, and there are some indications that the traditional “healthy immigrant effect” has weakened, reflecting evolving immigration patterns and an increase in immigrants who originate from conflict zones [14]. All these factors may influence self-reported life satisfaction.

The global literature from 117 countries in which life satisfaction scores of local born were compared to immigrants showed a high overall correlation of 0.96 [10]. Less is published regarding how life satisfaction is distributed across racialized groups and immigrants sometimes referred to as ‘visible minorities’ in high-income countries. The term *immigrant* denotes persons born outside Canada who are, or have ever been, landed immigrants/permanent residents or naturalized citizens. The term *racialized* denotes participants who, based on self-reported ethnicity identify with groups other than White and other than Indigenous peoples. In prior studies, racialized immigrants are more likely to experience a decline in self-reported health than non-racialized immigrants—a disparity that robust social networks can mitigate [15,16].

Few prior studies in Canada have investigated life satisfaction in relation to racialized and immigrant status, social disadvantage, discrimination, and healthcare status. To address this gap, we used the Cantril Ladder to assess life satisfaction among participants of the Canadian Alliance for Healthy Hearts and Minds (CAHHM), aiming to elucidate the determinants of life satisfaction among Canadian adults and testing the hypothesis that socially disadvantaged racialized immigrants experience the lowest levels [17].

## Materials and methods

### Ethics statement

Ethics approval was obtained from the Hamilton Integrated Research Ethics Board (HiREB #15277) and all relevant local ethics boards. All participants provided written informed consent.

### Study design and participants

This is a cross-sectional analysis of data which were obtained from the CAHHM cohort study, previously described [17]. Briefly, the CAHHM recruited adults from the general population between February 13, 2014, and February 28, 2018, and investigated health behaviours, contextual and socio-cultural factors, chronic disease risk factors, and completed a brain and cardiac MRI in all participants. The CAHHM population is currently undergoing repeat examinations 8 years from the baseline assessment. All participants provided written informed consent. Questionnaires were self-administered. CAHHM comprises 8,580 individuals (54% women) aged 30–78 years from communities across Canada.

### Measurement of life satisfaction

The primary outcome variable in this analysis: life satisfaction, was measured using the Cantril ladder score. This psychometrically validated scale offers a quantitative measure of subjective well-being, ranging from 0 (worst possible life) to 10 (best possible life) [2]. The Cantril Ladder has displayed reliability and validity in diverse adult populations. In a sample of U.S. adults, the past, present, and future ladder questions had a test-retest correlation ranging from 0.59-0.71 over a one month period, with highest reliability when referring to the present. [18,19] Validity has been shown in a pooled analysis of five studies involving 762 adults, in which the ladder correlated at 0.68 with the Satisfaction with Life Scale.[20] The Cantril ladder was selected over other fixed-scale measures because it allows respondents to self-anchor based on their perspective and has been widely utilized across diverse populations worldwide, as reflected in its inclusion in the Gallup World Happiness Report.[1,21] From this work in which hundreds of thousands of people have been surveyed from over 150 countries, three distinct and independent categories of life satisfaction were defined and labelled as suffering (0–4), satisfactory [5–6], and thriving [7–10].

### Measurement of sociodemographic variables/covariates

Demographic and lifestyle data were collected through standardized questionnaires. The information included demographic variables, cardiovascular risk factors (CVRFs), health behaviours, access to health care, and socioeconomic factors as previously described [17]. Ethnicity was self-reported, and from this information, racialized status (referring to non-white individuals) was derived to align with Canada’s “visible minority” framework. Physical activity [assessed by short form International Physical Activity Questionnaire (IPAQ-S)], psychological stress, income, employment, and education levels were also self-reported [17]. Social disadvantage was derived using an index predictive of CVD [22]. Healthcare access was assessed by the Health Services Questionnaire (HSR), which includes questions on access to care, health behaviours, cardiovascular risk factors, cardiac diagnostic testing, and information to calculate the non-lab INTERHEART risk score—a validated score for myocardial infarction risk [23,24]. Social support was defined as measured using questions about neighbours’ trust and neighbours helping to solve problems [17]. Participants in CAHHM who were immigrants completed the Vancouver Index of Acculturation (VIA) [25], which enables scoring to determine an individual’s affinity to their heritage culture and North American culture, years since immigration, and a specific immigrant questionnaire using questions from the Longitudinal Survey of Immigrants to Canada [26].

### Statistical considerations

Demographic and social characteristics were presented across strata based on immigration and racialized status in the overall population and based on racialized and experiencing discrimination status in the immigrant subpopulation. We analyzed the Cantril ladder (values 0–10) as a continuous measure, as this score is widely analyzed as such, yielding interpretable mean differences [27]. Univariable (not shown) and multivariable ordinary least squares linear regression models were used to estimate the impact of the variables on the Cantril score. We report β-coefficients as mean changes in ladder score. To build the final descriptive multivariable models, variables with a p < .20 in the univariable models and those which were of subject matter interest based on the literature were considered in a backward stepwise selection approach. Variables were reviewed for collinearity and in case of collinearity, the variable choice was based on model fit and clarity. A 2-sided p < .05 was considered nominally significant with no adjustment for multiple testing. All analyses were completed using SAS software, version 9.4 (SAS Institute Inc).

## Results

### Overall

We stratified participants into four groups based on racialized and immigrant status. The demographic characteristics of all four groups are presented in Table 1. Briefly, we observed the greatest differences in socio-demographic characteristics between non-racialized Canadian-born participants and racialized immigrants. For example, education level was higher amongst racialized immigrants compared to non-racialized Canadian-born participants, whereas age, annual income > $100, 000 Canadian dollars and cardiovascular risk scores were lower among racialized immigrants compared to non-racialized Canadian-born participants, as were factors such as language-concordance with family doctor, ease of seeing a medical specialist, and neighbourhood support (trust, collective problem solving, and willingness to help each other). Furthermore, there was a higher percentage of racialized immigrants who reported being unable to fill prescriptions due to cost compared to the other groups (Table 1 and Table 2).

**Table 1:** Baseline Demographic Characteristics of Participants Stratified by Immigration and Racialized Status.

	Non-racialized Canadian Born	Racialized Canadian Born	Non-racialized Immigrants	Racialized Immigrants
Number of participants	5612	309	955	1187
Female	3096 (55.2)	183 (59.2)	469 (49.1)	626 (52.7)
Age, mean (SD), y	58.1 (8.6)	53.9 (9.3)	60.2 (9.4)	56.1 (8.9)
**Highest Education Attained**				
High school or less	853 (15.5)	24 (8.0)	92 (9.7)	96 (8.3)
College or Trade	1883 (34.3)	83 (27.7)	276 (29.2)	305 (26.4)
University Degree	2754 (50.2)	193 (64.3)	576 (61.0)	753 (65.3)
**Employment**				
Full or part time	3801 (69.3)	238 (78.5)	629 (66.8)	808 (70.2)
Retired	1307 (23.8)	46 (15.2)	246 (26.1)	244 (21.2)
No paid work	377 (6.9)	19 (6.3)	67 (7.1)	99 (8.6)
Living with partner/married	4080 (74.3)	213 (70.3)	737 (78.0)	911 (79.6)
**Average Household Income**				
< $50,000	865 (15.4)	36 (11.7)	136 (14.2)	230 (19.4)
$50,000 - $74,999	944 (16.8)	48 (15.5)	180 (18.8)	196 (16.5)
$75,000 - $99,999	951 (16.9)	47 (15.2)	143 (15.0)	185 (15.6)
$100,000 - $149,999	1289 (23.0)	61 (19.7)	214 (22.4)	223 (18.8)
$150,000 or more	1144 (20.4)	85 (27.5)	210 (22.0)	217 (18.3)
Not captured	419 (7.5)	32 (10.4)	72 (7.5)	136 (11.5)

Presented data are n (%) unless otherwise specified.

**Table 2:** Baseline Social Characteristics of Participants Stratified by Immigration and Racialized Status.

	Non-racialized Canadian Born	Racialized Canadian Born	Non-racialized Immigrants	Racialized Immigrants
Number of participants	5612	309	955	1187
Social disadvantage index, mean (SD)	1.2 (1.3)	1.1 (1.3)	1.3 (1.3)	1.3 (1.3)
**Social disadvantage**				
Low disadvantage	3050 (54.3)	182 (58.9)	507 (53.1)	625 (52.7)
Mod disadvantage	1801 (32.1)	78 (25.2)	321 (33.6)	348 (29.3)
High disadvantage	331 (5.9)	17 (5.5)	52 (5.4)	71 (6.0)
Incomplete data for classification*	430 (7.7)	32 (10.4)	75 (7.9)	143 (12.0)
**Neighbours Questions**				
If a problem, neighbours work together to deal with it (agree)	4224 (75.6)	228 (74.0)	736 (77.8)	848 (71.7)
Neighbours trust each other	5224 (93.5)	276 (89.6)	880 (93.0)	1044 (88.3)
Neighbours willing to help their neighbours	5040 (90.1)	271 (87.7)	856 (90.1)	1029 (86.9)
INTERHEART risk score, mean (SD)	10.5 (6.0)	9.5 (5.6)	10.2 (5.9)	9.6 (5.4)
**INTERHEART Risk Score**				
Low	2689 (48.1)	157 (50.8)	484 (50.8)	637 (53.8)
Moderate	1793 (32.1)	106 (34.3)	307 (32.2)	387 (32.7)
High	1108 (19.8)	46 (14.9)	161 (16.9)	159 (13.4)
**Access to Health Services**				
Have a regular primary care provider	5317 (94.8)	293 (94.8)	910 (95.4)	1132 (95.5)
Visited primary care provider in past 12 months				
Yes	4803 (85.6)	243 (78.6)	824 (86.4)	1057 (89.2)
No or do not know	512 (9.1)	50 (16.2)	86 (9.0)	75 (6.3)
Do not have a primary care provider	293 (5.2)	16 (5.2)	44 (4.6)	53 (4.5)
Speak same language as primary care provider				
Yes	5283 (94.3)	292 (94.5)	893 (93.6)	1036 (87.5)
No	28 (0.5)	1 (0.3)	17 (1.8)	95 (8.0)
Do not have a primary care provider	293 (5.2)	16 (5.2)	44 (4.6)	53 (4.5)
Went to Emergency in past 12 months	907 (16.2)	38 (12.3)	139 (14.6)	150 (12.7)
Experience any difficulties getting the routine or ongoing care	618 (11.0)	31 (10.0)	104 (10.9)	123 (10.4)
Difficulties getting specialist care in past 12 months				
Yes	501 (8.9)	20 (6.5)	97 (10.2)	134 (11.3)
No or do not know	2582 (46.1)	127 (41.1)	460 (48.4)	497 (41.9)
Did not need specialist	2523 (45.0)	162 (52.4)	394 (41.4)	554 (46.8)
Did not fill prescription because of the cost	144 (2.6)	13 (4.2)	27 (2.8)	64 (5.4)
Cantril Ladder Score, mean (SD)	7.2 (1.4)	7.2 (1.5)	7.2 (1.5)	6.6 (1.6)
**Cantril Categories**				
Suffering [1–4]	243 (4.3)	17 (5.5)	42 (4.4)	105 (8.8)
Struggling [5–6]	1270 (22.6)	61 (19.7)	229 (24.0)	402 (33.9)
Thriving [7–10]	4099 (73.0)	231 (74.8)	684 (71.6)	680 (57.3)

Presented data are n (%) unless otherwise specified. *The social disadvantage index is based on self-reported income, employment and marital status. We respect that some participants chose to skip some responses possibly due to perceived stigmatism, yet we wanted to make sure our analyses reflected the other responses of this same group.

**Fig 1:**
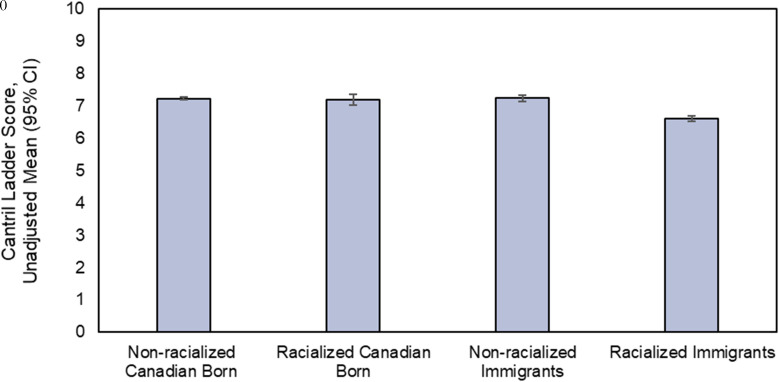
Life satisfaction scores across demographic groups (adjusted for years in Canada). P for differences between groups <0.0001.

The life satisfaction scores were lowest among racialized immigrants compared to all other groups, using non-racialized Canadian-born participants as the reference group (6.6 ± 1.6 vs. 7.2 ± 1.4), P < 0.0001). These differences between racialized immigrants and non-racialized Canadian-born participants persisted after adjustment for years of living in Canada [6.8 (95% CI: 6.7-6.9) vs. 7.2 (95% CI: 7.1-7.2)] (Fig 1). Racialized immigrants compared to non-racialized Canadian-born participants were less likely to report a ladder score in the thriving range of 7–10 (57.3 vs. 73.0%), and more likely to report scores in the struggling range of 5–6 (33.9 vs. 22.6%) or in the suffering range of 0–4 (8.8 vs. 4.3%), respectively (Table 2).

**Fig 2:**
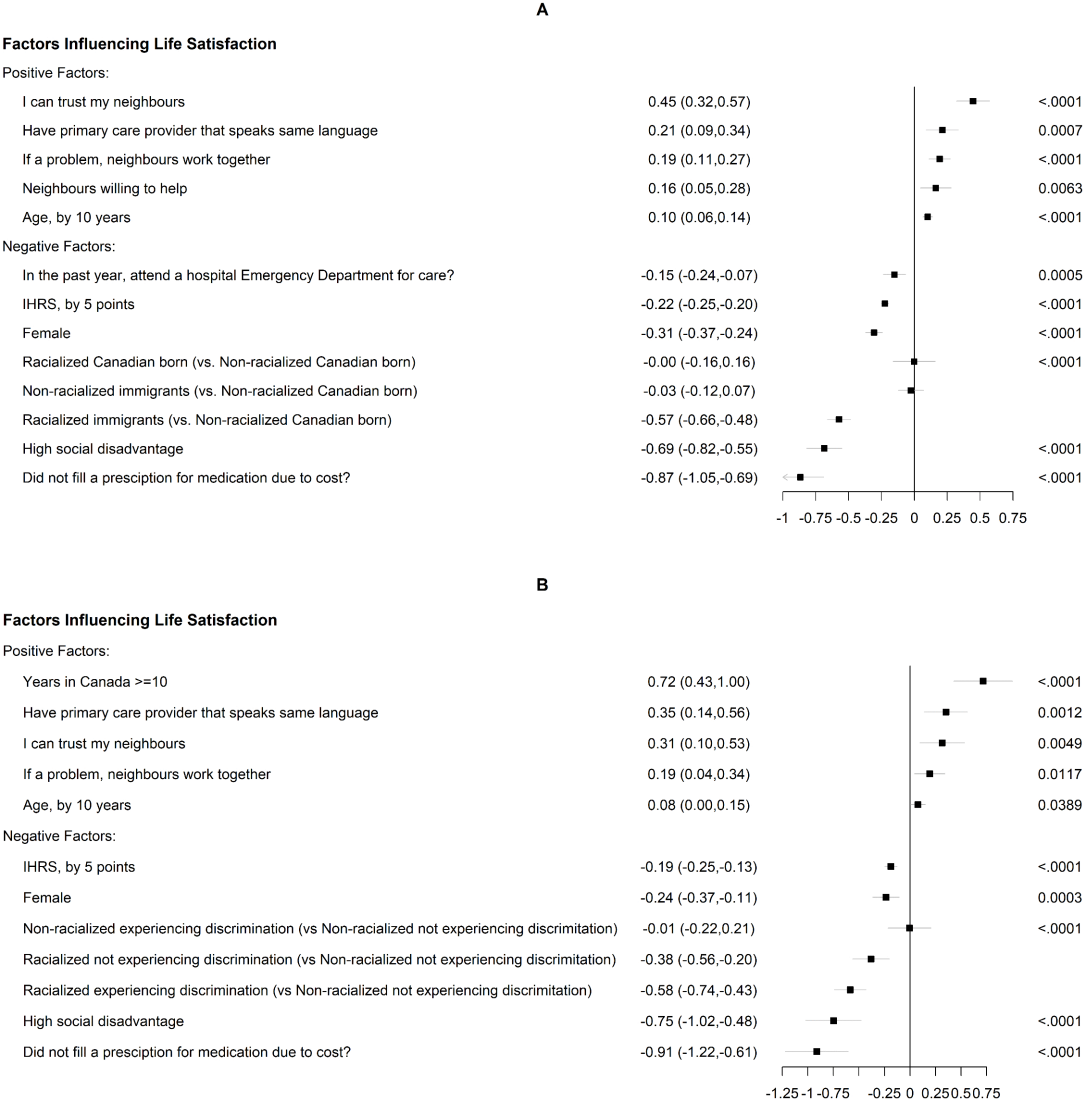
(a) Determinants of life satisfaction scores in the overall cohort. (b) Determinants of life satisfaction scores in the immigrant cohort. *IHRS = INTERHEART Risk Score.

The factors associated with life satisfaction in all participants are shown in Fig 2a. Briefly, in order of magnitude, factors related to lower life satisfaction included not being able to fill a prescription due to cost, social disadvantage, being an racialized immigrant, female sex, the presence of CVRFs, and having had an emergency room visit in the last 12 months. Factors related to improved life satisfaction included having trusted neighbours, having a language-concordant primary care doctor, having neighbours to help solve problems, and increasing age (Fig 2a and Table 3).

**Table 3:** Predictors of the Cantril Score (continuous) in all participants (N = 7970).

Variable	Change in Cantril 95% CI	P-value F-test
**Racialization and Immigration Status, Non-Racialized Canadian Born (Reference Group)**		
Racialized Canadian Born	-0.00 (-0.16,0.16)	<.0001
Non-racialized Immigrants	-0.03 (-0.12,0.07)	.
Racialized Immigrants	-0.57 (-0.66, -0.48)	.
Age, by 10 years	0.10 (0.06,0.14)	<.0001
Female	-0.31 (-0.37, -0.24)	<.0001
High social disadvantage (SDI)	-0.69 (-0.82, -0.55)	<.0001
If a problem, neighbours work together Y vs N	0.19 (0.11,0.27)	<.0001
Neighbours willing to help Y vs N	0.16 (0.05,0.28)	0.0063
I can trust my neighbours Y vs N	0.45 (0.32,0.57)	<.0001
INTERHEART Risk** **Score by 5 points	-0.22 (-0.25, -0.20)	<.0001
Have primary care AND speak same language (vs no primary care OR doesn’t speak same language)	0.21 (0.09,0.34)	0.0007
In the past 12 months, did you go to a hospital Emergency Department for care?	-0.15 (-0.24, -0.07)	0.0005
Did not fill a prescription for medication because of the cost?	-0.87 (-1.05, -0.69)	<0.0001

Estimates from multivariable linear regression

Legend: Y = Yes, N= No, SDI: Social disadvantage index, higher equals greater disadvantage

### Immigrant subset

Amongst immigrants, questions regarding discrimination were collected as part of the acculturation questionnaire (S1 Table). Among 2,142 who completed these questions, racialized immigrants reported experiencing more discrimination compared to non-racialized immigrants (64 vs. 27%, respectively). The discrimination based on skin colour (as a proportion of the ethnic group) was largely driven by the experience of Black participants (i.e., 67% in Black people vs. 37% South Asians, 34% Chinese, and 30% in the other immigrant category). The most reported reasons for discrimination for both racialized and non-racialized are shown in S2 Fig. Other differences between racialized immigrants vs. non-racialized immigrants included their lower affinity to North American culture and their shorter duration of time living in Canada, which was, on average 10 years less. (S3 Fig)

Amongst immigrants in the multivariate regression model, factors related to lower life satisfaction in order of magnitude included: not being able to fill a prescription due to cost, high social disadvantage, experiencing discrimination, being racialized (even if they did not report discrimination), female sex, and having cardiovascular risk factors. Factors related to higher life satisfaction included years lived in Canada, having a language-concordant primary care doctor, having trusted neighbours and older age (Fig 2b and Table 4).

**Table 4:** Predictors of Cantril Score (continuous) in Immigrant Subpopulation (N = 2013).

Variable	Change in Cantril 95% CI	P-value F-test
**Racialization and Discrimination,** Non-Racialized Not Experiencing Discrimination (Reference Group)		
Racialized Not Experiencing Discrimination	-0.38 (-0.56, -0.20)	<.0001
Non-racialized Experiencing Discrimination	-0.01 (-0.22,0.21)	.
Racialized Experiencing Discrimination	-0.58 (-0.74, -0.43)	.
Age, by 10 years	0.08 (0.00,0.15)	0.039
Female	-0.24 (-0.37, -0.11)	0.0003
High social disadvantage (SDI)	-0.75 (-1.02, -0.48)	<.0001
If a problem, neighbours work together Y vs N	0.19 (0.04,0.34)	0.012
I can trust my neighbours Y vs N	0.31 (0.10,0.53)	0.005
INTERHEART Risk Score by 5 points	-0.19 (-0.25, -0.13)	<.0001
Have primary care AND speak same lang (vs no primary care OR doesn’t speak same language)	0.35 (0.14,0.56)	0.001
Did not fill a prescription for medication because of the cost?	-0.91 (-1.22, -0.61)	<.0001
Years in Canada >=10	0.72 (0.43,1.00)	<.0001

Estimates from multivariable linear regression

Legend: Y = Yes, N= No, SDI: Social disadvantage index, higher equals greater disadvantage

## Discussion

This cross-sectional analysis of 8,063 Canadian adults reveals that while overall life satisfaction is relatively high, racialized immigrants report significantly lower scores especially those experiencing discrimination. Key determinants of lower life satisfaction include financial hardship (specifically the inability to afford prescription medications), higher social disadvantage, being female, and increased cardiovascular risk factors. In contrast, protective factors such as trustworthy neighbours, having a language-concordant family doctor, older age, and longer residence in Canada buffer these negative effects.

Health status and access play a critical role in life satisfaction. The inability to afford prescription medications – indicative of financial strain was the strongest determinant of lower life satisfaction. This issue is compounded by provincial disparities in drug coverage and by Canada’s lack of a national pharmacare plan, making it the only country without universal prescription coverage but with universal health care [28]. Our findings support renewed calls for universal pharmacare as a mechanism to reduce health inequities, improve medication adherence and ultimately to enhance well-being. Continuous primary-care attachment is associated with lower emergency-department reliance, and provider–patient language concordance is independently associated with higher life satisfaction among those whose first language is not English (or French). While Canada currently has a shortage of primary care providers, in the rebuilding of the primary care systems consideration to language concordance is key.

Structural inequalities persist, particularly for racialized groups, where both financial constraints and healthcare access contribute to lower satisfaction. Addressing these disparities through national pharmacare and improving language-concordant care may help mitigate these issues. Social disadvantage, including employment and housing instability, remains a powerful determinant in life satisfaction, emphasizing the need for targeted policy interventions. Additional adverse factors include recent emergency room visits and cardiovascular risk burdens, while language-concordant care is associated with improved outcomes. Structural racism leads to greater social disadvantage and lower health status. Our findings, consistent with prior studies [29–31], indicate that racialized individuals experience higher levels of discrimination in healthcare, contributing to their lower life satisfaction. Moreover, our analysis shows that immigrants tend to have a stronger affinity for their heritage culture, whereas Canadian-born individuals exhibit a greater affinity for Canadian culture. This cultural alignment may further influence their overall well-being. Health systems should collect and report race-/ethnicity-disaggregated patient-reported experience measures, implement inclusion quality indicators (e.g., respectful communication, interpreter offer/uptake), and fund community partnerships that build neighbourhood trust and social capital [32].

Our observation that racialized immigrants have significantly lower life satisfaction scores and lower report of “thriving” compared to other Canadians suggests that policies and programs which lead to greater integration and inclusion of newcomers to Canada are needed. While settlement services are most developed for refugees (e.g., private sponsorship “Group of Five”), family- and economic-class immigrants often have limited access to comparable supports beyond initial orientation [33]. Programs that generalize the effective elements of refugee sponsorship—navigation, rapid primary-care attachment, language support, and community linkages—to other immigrant streams are likely to yield well-being gains, particularly among racialized newcomers. Immigrants to Canada can come for family or economic reasons, or as refugees [10]. The “Group of Five” model illustrates how structured community support can accelerate access to housing, primary care, schooling, and social networks, thereby building trust and reducing early stressors [33]. Analogous mechanisms—primary-care attachment with interpretation/language concordance and reduction of medicine cost barriers—map directly onto the strongest determinants observed in our analysis.

Strengths of this study include its large, multi-ethnic sample and the use of the validated Cantril Ladder to assess life satisfaction. A unique contribution of this study is the joint examination of immigration status, racialization, and self-reported discrimination within a large, national dataset that integrates both health (cardiometabolic risk, health status, care access) and social variables (affordability, neighbourhood trust, language concordance). However, the cross-sectional design limits causal interpretations and residual confounding may persist despite adjustments. Nevertheless, the CAHHM sample—older, more educated, and with lower cardiovascular risk than the general population [23]—likely biases associations toward the null, meaning the true inequities in life satisfaction in the broader Canadian population are probably larger than observed here. An additional limitation of our analysis was discrimination was only assessed amongst the immigrant subset as they completed the “immigrant questionnaire” based on the Longitudinal Survey of Immigrants to Canada [26], thus non-immigrant participants did not have discrimination assessed. Future longitudinal analyses will be essential to better understand the temporal relationships between these factors and life satisfaction. Priority next steps include prospective analyses within CAHHM, linkage to administrative and pharmacy claims to quantify cost-related nonadherence, and policy evaluations (e.g., quasi-experimental assessment of pharmacare or primary-care attachment reforms) to determine causal impacts on life satisfaction.

## Conclusions

Our comprehensive analysis demonstrates that high national life-satisfaction averages in Canada mask inequities by race and immigration status. Complex interactions among social, demographic, cardiometabolic, and healthcare factors shape subjective well-being, with racialized immigrants reporting lower life satisfaction and a lower likelihood of “thriving.” We identify modifiable levers—language-concordant primary care, neighbourhood trust, affordable medications to reduce cost-related nonadherence, and improved cardiometabolic health—that support immediate health-system and policy action.

## Supporting information

S1 Fig Graphical abstract. (TIF)

S2 Fig Commonly reported reasons for discrimination among Immigrants. (TIF)

S3 Fig Immigrant years in Canada by racialized status. (TIF)

S1 Table Baseline Characteristics for Immigrant Subpopulation Specific Measures. (DOCX)
