# Supplementary figures and images for: Who thrives in Canada? An Examination of social factors, healthcare access, and immigration status
Source: PLOS Glob Public Health. 2025 Dec 4;5(12):e0005257. doi: 10.1371/journal.pgph.0005257 (PMC12677554; doi:10.1371/journal.pgph.0005257)

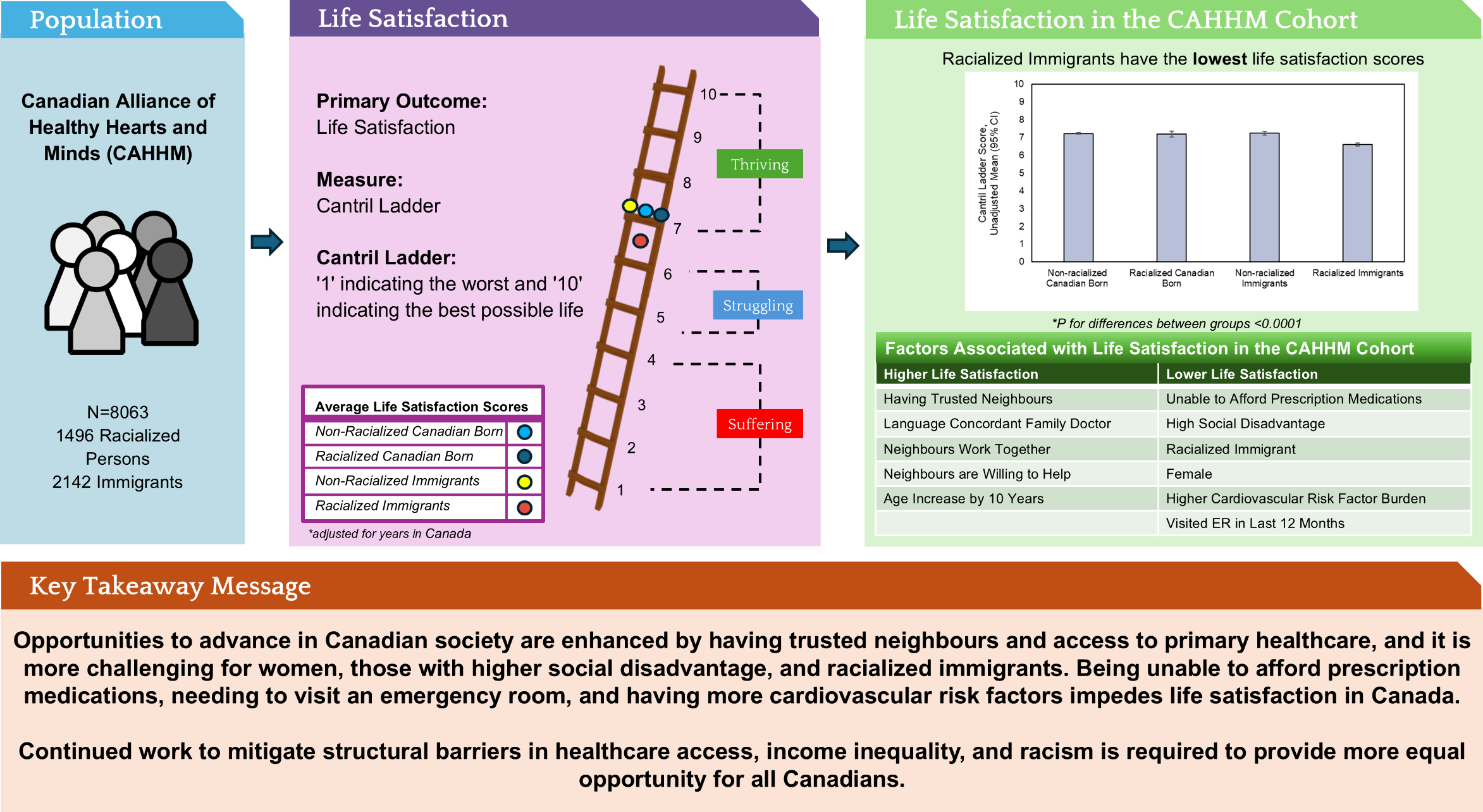

Supplement: S1 Fig — (TIF) [file pgph.0005257.s001.tif]

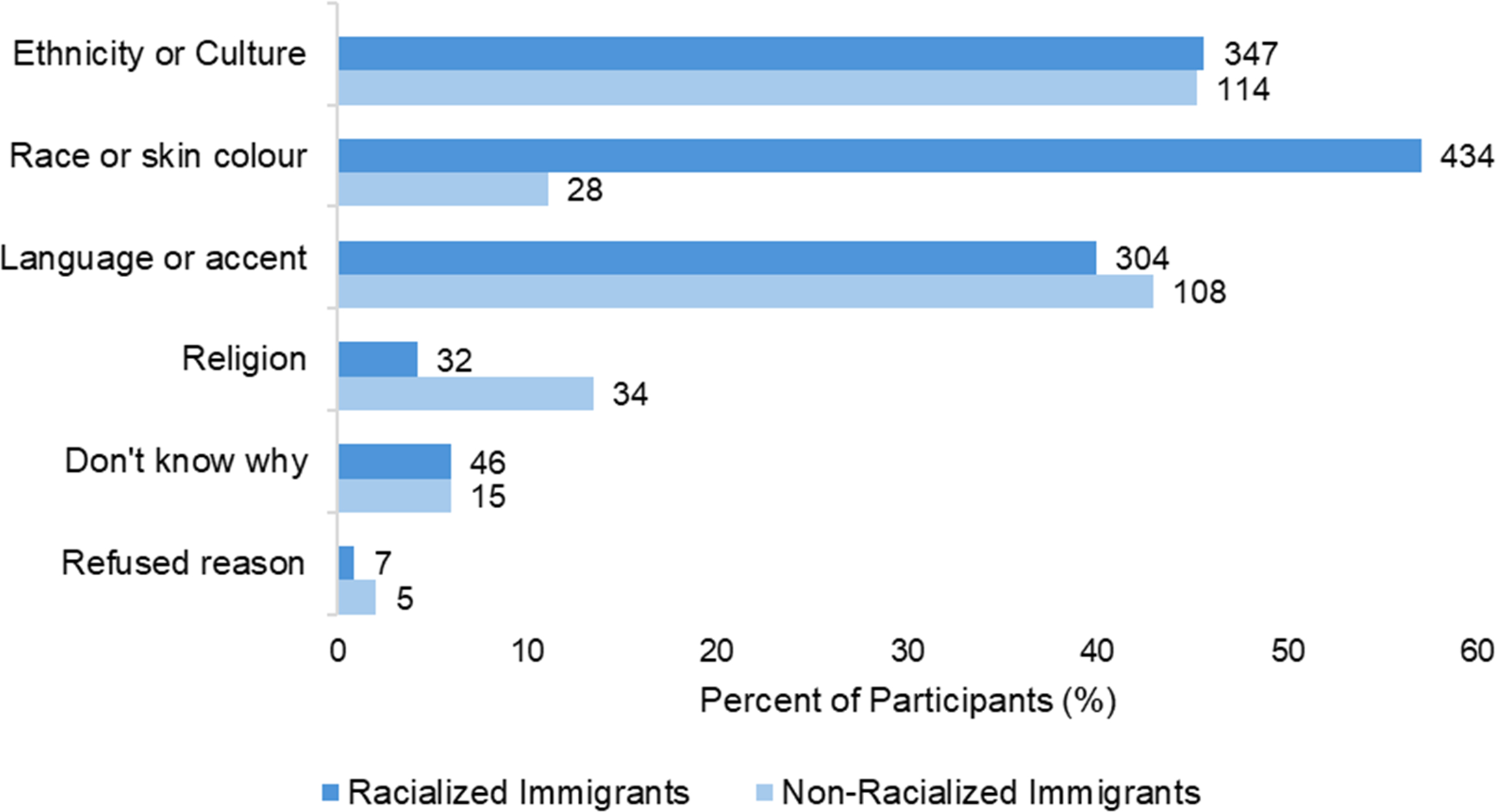

Supplement: S2 Fig — (TIF) [file pgph.0005257.s002.tif]

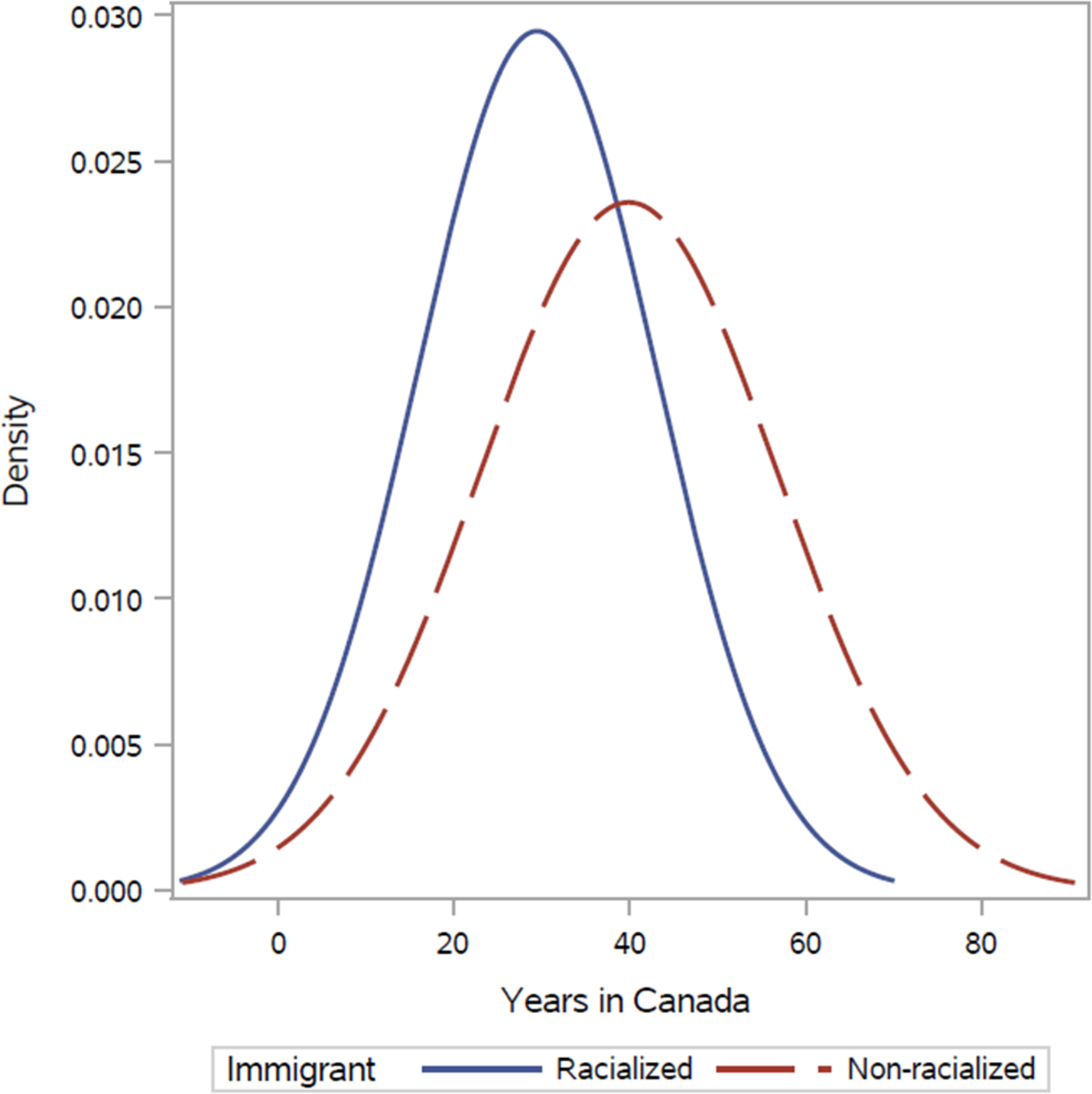

Supplement: S3 Fig — (TIF) [file pgph.0005257.s003.tif]
