# Supplementary material for: Who thrives in Canada? An Examination of social factors, healthcare access, and immigration status
Source: PLOS Glob Public Health. 2025 Dec 4;5(12):e0005257. doi: 10.1371/journal.pgph.0005257 (PMC12677554; doi:10.1371/journal.pgph.0005257)
Supplement: S1 Table — (DOCX) [file pgph.0005257.s004.docx]

**Table S1. Baseline Characteristics for Immigrant Subpopulation Specific Measures**

|  | **All Immigrants to Canada** | **Racialized Immigrants** | **Non-Racialized Immigrants** |
| --- | --- | --- | --- |
| Number of participants | 2142 | 1187 | 955 |
| Have you experienced discrimination or been treated unfairly by others | 1013 (47.5) | 761 (64.3)* | 252 (26.5) |
| Reasons^1^ for experienced discrimination |  |  |  |
| Ethnicity or Culture | 461 (45.5) | 347 (45.6) | 114 (45.2) |
| Race or skin colour | 462 (45.6) | 434 (57.0)* | 28 (11.1) |
| Language or accent | 412 (40.7) | 304 (39.9) | 108 (42.9) |
| Religion | 66 (6.5) | 32 (4.2)* | 34 (13.5) |
| Refused reason | 12 (1.2) | 7 (0.9) | 5 (2.0) |
| Don't know why | 61 (6.0) | 46 (6.0) | 15 (6.0) |
| VIA, mean (SD) |  |  |  |
| VIA-Heritage affinity | 6.4 (1.3) | 6.4 (1.3)* | 6.3 (1.4) |
| VIA-North American affinity | 6.5 (1.3) | 6.2 (1.2)* | 6.9 (1.2) |
| **How long have you lived in Canada, mean (SD)** | 34.1 (16.0) | 29.5 (13.5)* | 39.9 (16.9) |
| < 10 years | 112 (5.2) | 86 (7.2) | 26 (2.7) |
| 10 - 19 years | 374 (17.5) | 232 (19.5) | 142 (14.9) |
| 20 - 29 years | 436 (20.4) | 321 (27.0) | 115 (12.0) |
| 30 - 39 years | 316 (14.8) | 197 (16.6) | 119 (12.5) |
| 40 - 49 years | 556 (26.0) | 289 (24.3) | 267 (28.0) |
| 50 - 59 years | 205 (9.6) | 51 (4.3) | 154 (16.1) |
| 60+ years | 143 (6.7) | 11 (0.9) | 132 (13.8) |

Presented data are n (%) unless otherwise specified.

^1^Multiple responses possible. We respect each reason selected including “Refused” and “Don’t know why”.

*p<.05 comparing racialized and non-racialized immigrants.

VIA = Vancouver Index of Acculturation
